# Critical Upper Limb Ischemia Due to Brachial Tourniquet in Misdiagnosed Thoracic Outlet Syndrome after Carpal Tunnel Decompression: A Case Report

**Published:** 2017-09

**Authors:** Cesare Tiengo, Andrea Monticelli, Stefano Bonvini, Valentina Wassermann, Erica Dalla Venezia, Franco Bassetto

**Affiliations:** 1 Plastic and Reconstructive Surgery Unit, Padova University Hospital, Padova, Italy;; 2 Vascular and Endovascular Surgery Unit, Padova University Hospital, Padova, Italy

**Keywords:** Thoracic outlet syndrome, Ischemia, Carpal tunnel syndrome, Complication

## Abstract

We present the case of a 68-year-old woman, referred to our department for critical upper limb ischemia, which had occurred a few days after homolateral surgical ligamentotomy for carpal tunnel syndrome, diagnosed and confirmed by electromyography, and performed with a brachial tourniquet. The patient was later admitted for subsequent progressive necrosis of the first three fingers of the left hand, accompanied by signs of upper limb ischemia. An accessory cervical rib was identified, completely obliterating the subclavian artery distally at the origin of the suprascapular artery. A complete humeral artery occlusion was also found at the middle third of the humerus. The accessory rib was resected and the subclavian artery recanalized. A few days later, necrosis of the distal third of the first two fingers appeared and surgical resection was performed. Despite this chronic condition, the acute occlusion of collateral circles was probably induced by the brachial tourniquet. This represents a rare event, never previously reported in the literature: a case of critical upper limb ischemia due to a brachial tourniquet in a patient with misdiagnosed thoracic outlet syndrome. Until specific electrophysiological criteria for this syndrome can be found, attention should focus on history and clinical examination in patients with suspected carpal tunnel syndrome.

## INTRODUCTION

Carpal tunnel syndrome (CTS) is the most common peripheral neuropathy,^1^ with a prevalence of 3.8% in the general population.^2^ Proximal dysfunctions seem to elevate the incidence of CTS, increasing the susceptibility of distal nerves. Several other disorders, such as narrowed cervical foramina and decreased cervical range of motion,^1^ have also been associated with CTS. However, its statistical association with Thoracic Outlet Syndrome (TOS) is still unclear in the literature. In these cases, diagnosis, defined by the association between clinical symptoms, physical examinations and electrophysiological studies, may turn out to be difficult and even insidious, and further examinations should be considered prior to surgery. We report here a case of a woman in whom an inaccurate diagnosis and subsequent surgical therapy resulted in severe consequences for her hand. This very unusual case induced us to reflect on the possible reasons of the unexplained failure of surgical treatment for CTS.

## CASE REPORT

We present the case of a 68-year-old woman, referred to our department for critical upper limb ischemia, which had occurred a few days after homolateral surgical ligamentotomy, after diagnosis of Carpal Tunnel Syndrome (CTS). A few months earlier, the patient had complained of pain, numbness and paresthesia in the left hand, affecting in particular the first three fingers at night. She had no history of cardiovascular diseases, arteriopathy, diabetes, smoking, or other vascular risk factors. 

She was clinically diagnosed with CTS, which was confirmed by electromyography of the left upper limb. A few weeks later, according to clinical and electrophysiological findings, surgical ligamentotomy was performed with a brachial tourniquet at a small local hospital. No complications occurred during the operation or after it, and the patient was sent home. The next day she returned to the hospital, complaining of extreme pain affecting the whole arm, but this was judged as wound pain and was medicated with painkillers. 

The following day she again returned to the emergency department, with the same incoercible pain, together with initial necrosis of the first three fingers of the left hand. She was immediately referred to the department of vascular surgery at the reference hospital. Clinical examination revealed delayed acute limb ischemia, in the absence of brachial pulsatility. A thoracic and upper limb angio-TC was immediately performed, and showed complete thrombotic occlusion of the left axillary artery and the first tract of the brachial artery with its distal rehabitlitation. 

**Fig 1:**
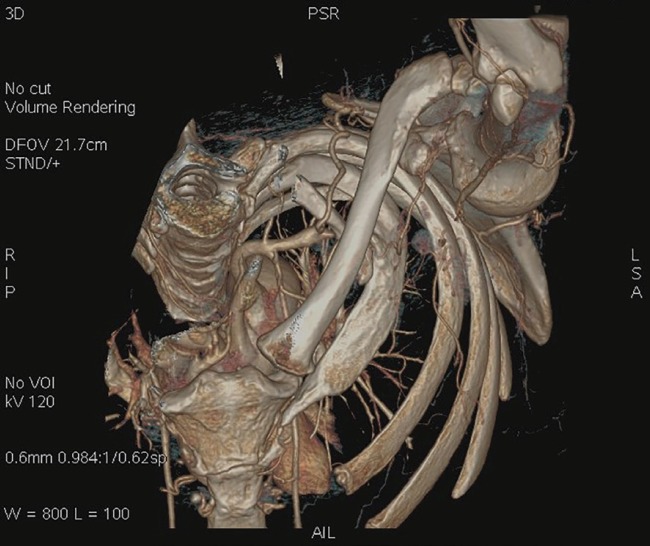
Preoperative cervicothoracic TC 3D reconstruction. The left accessory rib was located in the costoclavicular space obliterating completely the subclavian artery. The suprascapular artery was hyperthrophic and collateral circles was formed

**Fig 2:**
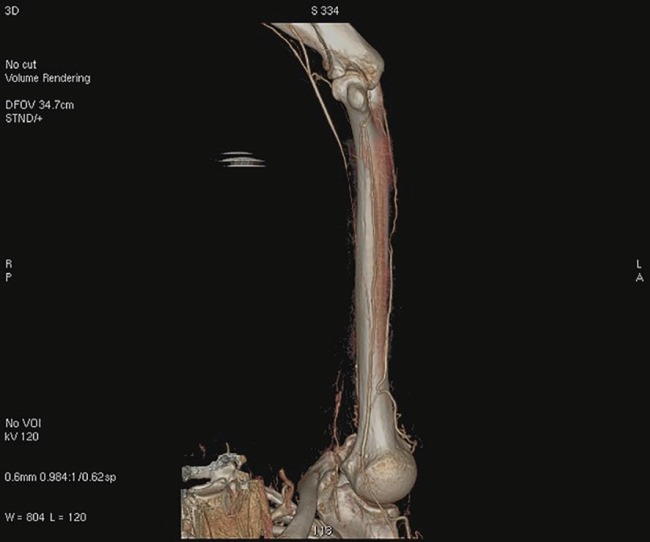
Preoperative cervicothoracic TC 3D reconstruction. Left brachial artery occlusion, compatible with the position of the brachial tourniquet

An accessory cervical rib was found, completely obliterating the left subclavian artery distal to the origin of the costocervical trunk (Figure 1) and a post-stenotic dilatation (aneurysm) was found. This condition did not appear to be new, as many secondary and collateral circles originating from the axillary artery had completely altered normal vascular anatomy. An obstruction of the left brachial artery was found at the middle third of the arm, in exactly the same position occupied by the brachial tourniquet during surgery (Figure 2). The vascular surgery team resected the accessory rib and performed thrombectomy of the brachial and axillary arteries with a Fogarty catheter. 

**Fig 3:**
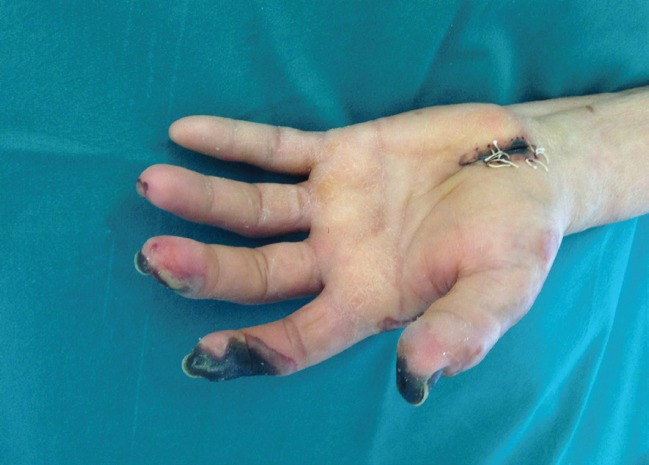
Preoperative left hand clinical situation: the vascular occlusion caused the necrosis of the first three fingers and the skin of the surgical wound

Due to the persistent absence of a brachial pulse, caused by dissection of the brachial artery, a venous graft was created, consisting of a left subclavian-proximal brachial artery bypass. At the end of the surgical procedure, a radial pulse was perceptible. Prescribed medical treatment included antiplatelet and anticoagulant therapy. After the first surgical treatment, complete necrosis of the distal third of the first two fingers became marked and a surgical resection was performed in the department of Plastic and Reconstructive Surgery (Figure 3). 

**Fig 4:**
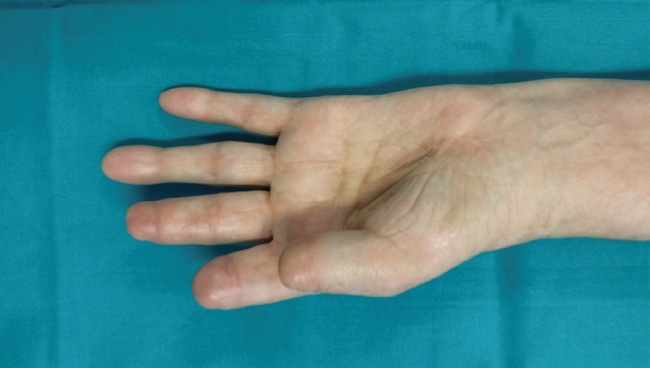
Ten months of follow-up. All wounds have been healed, no subsequent complications have been occurred

In the post-operative period, an eco-color Doppler demonstrated the patency of the venous graft. Currently, the patient does not complain of any pain, her pulse is clinically perceptible, the temperature of her hand is normal, and the wounds have healed (Figure 4). The patient and her family were informed that her case was of interest for publication, and their written consent was obtained.

## DISCUSSION

Thoracic Outlet Syndrome (TOS) is now defined as a group of clinical disorders involving suffering of the brachial plexus and subclavian vessels at their thoracic outlet.^1^^,^^3^ It may be caused by compression between the first rib and the clavicle (costoclavicular syndrome, the most common cause of arterial abnormalities in TOS),^4^ between the costoclavicular space and the thoraco-coraco-pectoral space during hyperabduction of the arms (hyperabduction syndrome), and at the narrowing of the interscalene triangle (scalenus anticus, cervical rib and TOS).^1^^,^^5^

Post-traumatic compression of neurovascular structures between the clavicle and the first rib have been described in the literature.^3^ A case of undiagnosed TOS, complicated by acute ischemia of the upper arm after surgical treatment for CTS in a 68-year-old woman, is described here. However, in our patient, the original diagnosis of CTS may have been correct. Proximal anatomic abnormalities may in fact increase the susceptibility of peripheral nerves, resulting in symptoms such as peripheral compression of the ulnar, radial and median nerves.^1^

This situation is called “double crush syndrome”, a term coined in 1973 by Upton and McComas,^6^ indicating two points of compression of the nerves of the upper limbs. Proximal neural damage probably disturbs normal axoplasmic flow and thus the metabolism of peripheral nerves.^5^ This syndrome may be mistakenly identified as CTS more often than distal compressive disease.^6^^,^^7^ Clinically revealed CTS has been found in varying percentages of patients affected by TOS, from 44%^6,8^ to nearly 0%,^8^ demonstrating the controversy and difficulty of diagnosing clinical TOS correctly.^6^

In addition, in two studies,^6^^,^^9^ no appreciable statistic link was found between CTS and true TOS: the frequency of proximal dysfunction in women affected by CTS was found to be less than 1%.^6^ True TOS is generally unilateral, with a history of paresthesia and pain starting in the supraclavicular fossa and irradiating through the medial arm, forearm and hand, especially in areas innervated by medial antebrachial cutaneous and ulnar nerves. Weakness, followed by atrophy, affects the thenar eminence and occasionally the medial forearm.^3^

Numbness and tingling are found in about 50% of patients, sometimes accompanied by sensory loss. Neurological and vascular symptoms are usually related to a post-stenotic aneurysm of the subclavian artery. This has been reported in 85% and 96% of patients with vascular abnormalities, respectively by Criado and Durham,^4^ and was also found in our case. Symptoms may be caused by specific maneuvers, such as postural exacerbation.^9^ Four provocative tests (timed Morley test, Wright test, Eden test, elevated arm stress exercise test)^5^ have been proposed to diagnose TOS, but they are of low specificity.^9^

Vascular examination is also very helpful, but it must be performed at an angle of abduction and external rotation not exceeding 90°, this point being essential to maintain good test specificity and reliability. Several studies have found modifications of the radial pulse during abduction and external rotation of the arm, especially in women, with higher clinical values than with Doppler ultrasound.^9^ Our patient was referred to us when a clearly defined vascular impairment appeared and diagnosis was apparently easy. No data regarding vascular or nervous symptoms or signs were reported when she went to her general practitioner, nor whether a complete examination was performed, but the patient stated that the only symptom was peripheral paresthesia in median territory.

Neurophysiological tests in patients with suspected TOS generally involve examination of the sensory nerve amplitude potential (SNAP) of the medial antebrachial cutaneous nerve (MABCN), which has been used to identify true neurogenic TOS since 1993.^9^ Our patient had also had electromyography when the first symptoms appeared, and they suggested CTS, as did the clinical examination. X-ray examination is essential for proper views of anomalous osseous structures, whereas MRI is more reliable in identifying fibrous bands at the thoracic outlet. Surgical treatment should not be based only on radiological findings, but also associated with clinical and EMG results.^3^

TOS due to an accessory rib is treated with surgical resection of the rib and, if necessary, revascularization of the occluded artery, as in our case. Several surgical approaches have been proposed in the literature; resection of anatomical abnormalities is always suggested when possible, but first rib resection is also often performed in these patients and is considered to be safe, although discomfort and morbidity may be substantial. The results of surgical treatments are difficult to evaluate and considerable heterogeneity is reported in the literature. Surgical indications are progressive neurological deficit and failure of conservative treatments.^3^

In our patient, resection of the accessory rib and revascularization of the left brachial artery by subclavian-brachial artery bypass interrupted further necrosis and wound healing. Despite the patient’s chronic condition, as we first stated, acute occlusion of the collateral circles was probably caused by the brachial tourniquet during surgery. Although spontaneous necrosis of the distal part of the upper limb in TOS patients has occasionally been reported, we could not find a similar situation in the literature. 

We believe our case represents “the tip of an iceberg” of unclear diagnoses of CTS, which subsequent surgical treatment cannot necessarily resolve. Since diagnosis of CTS is clinical, the surgeon must identify suspicious cases and perform specific clinical tests. In addition, if suspicion is confirmed or remains unclear, electromyography should be carried out in the specific hypothesis of TOS; otherwise, only CTS may be identified if a non-specific question has been made. In order to complete the diagnosis, radiological examinations should subsequently be carried out.

## CONFLICT OF INTEREST

The authors declare no conflict of interest.
